# Novel *NLRC4‐ALK* and *EML4‐ALK* double fusion mutations in a lung adenocarcinoma patient: A case report

**DOI:** 10.1111/1759-7714.13389

**Published:** 2020-03-24

**Authors:** Xueqian Wu, Weiya Wang, Bingwen Zou, Yanying Li, Xiaojuan Yang, Ning Liu, Qizhi Ma, Xiaoxuan Zhang, Yongsheng Wang, Dan Li

**Affiliations:** ^1^ Department of Thoracic Oncology, State Key Laboratory of Biotherapy and Cancer Center, West China Hospital Sichuan University and Collaborative Innovation Center Chengdu China; ^2^ Department of Pathology, West China Hospital Sichuan University Chengdu China; ^3^ Department of Oncology The Second Affiliated Hospital of Chongqing Medical University Chongqing China; ^4^ Institute of Drug Clinical Trial, West China Hospital Sichuan University Chengdu China; ^5^ Precision Medicine Center, Precision Medicine Key Laboratory of Sichuan Province, West China Hospital Sichuan University Chengdu China

**Keywords:** Crizotinib, double *ALK* fusions, *EML4‐ALK*, lung adenocarcinoma, *NLRC4‐ALK*

## Abstract

Anaplastic lymphoma kinase (*ALK*) rearrangements have been reported in 5% to 6% of non‐small cell lung cancer (NSCLC) patients. However, the concurrent existence of two *ALK* fusions within the same patient have rarely previously been reported. Moreover, considering the diversities of *ALK* mutations, it is necessary to evaluate the response of both double and new types of *ALK* fusions to ALK‐tyrosine kinase inhibitors (ALK‐TKIs). Here, we report a case of a 64‐year‐old Chinese woman who was diagnosed with lung adenocarcinoma (ADC) who concurrently harbored two types of *ALK*‐rearrangements, including an unreported *NLRC4‐ALK* fusion and *EML4‐ALK* fusion. After surgery, the patient had a progression‐free survival (PFS) of over 10 months with continuous crizotinib treatment after surgery. Our findings provide a better understanding of ALK‐TKI in patients with two novel *ALK* concomitant fusions.

**Key points:**

A lung adenocarcinoma patient harboring concurrent *NLRC4‐ALK* and *EML4‐ALK* fusion mutations benefited from crizotinib after surgery. Our findings provide important information for future treatment decision‐making in patients with double *ALK* fusions.

## Introduction

Non‐small‐cell lung cancer (NSCLC) has been estimated to account for 80% to 85% of the total number of lung cancers.^1^ Anaplastic lymphoma kinase (*ALK*) gene rearrangements have been reported in 5% to 6% of NSCLC patients, especially in light or non‐smokers.^2^ So far, more than 30 types of *ALK* fusion partners (such as *EML4*, *KIF5B* and *KLC1*) have been identified in NSCLC.^3^ Crizotinib, a first‐generation ALK‐TKI, has been recommended as a first‐line therapy for *ALK*‐rearranged NSCLC, and has shown impressive single‐agent activity in *ALK*‐positive lung adenocarcinoma (ADC).^4^ Second‐generation (alectinib, ceritinib, and brigatinib) and third‐generation (lorlatinib) of ALK‐TKIs have also been developed.^5^ In this report, we present for the first time an unreported *NLRC4‐ALK* fusion mutation concurrently with *EML4‐ALK* in an ADC patient.

## Case report

**Figure 1:**
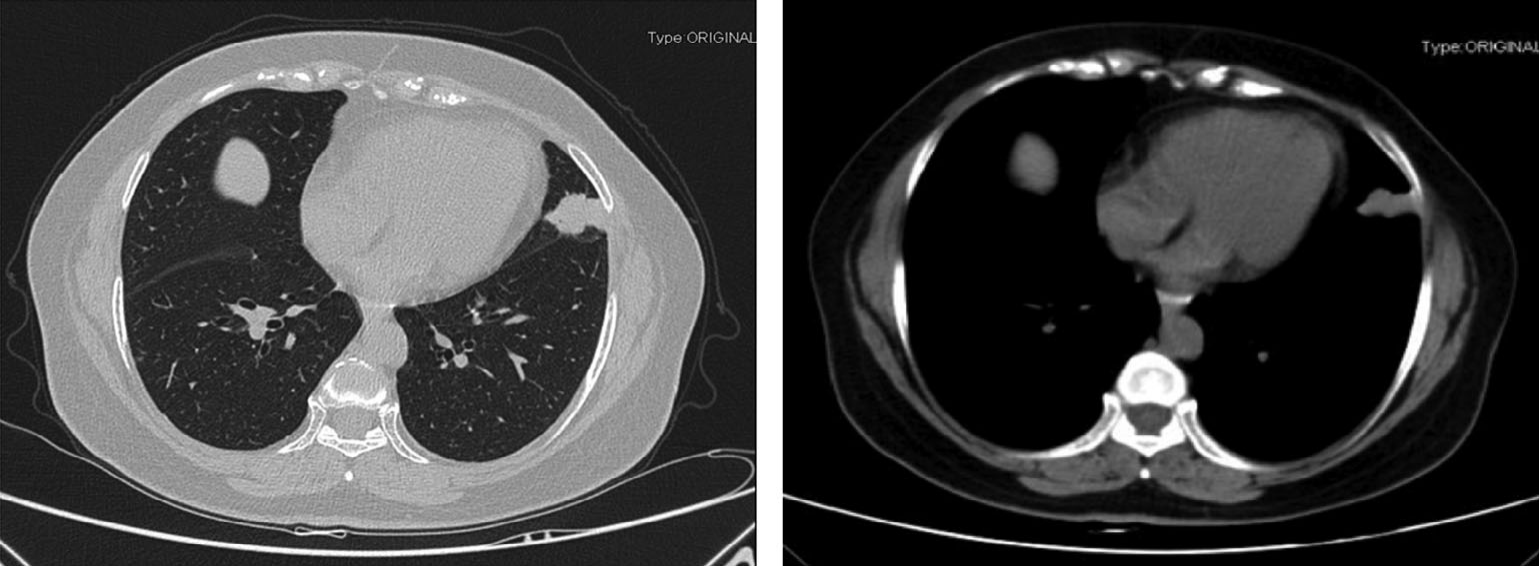
Computed tomography (CT) scan showed the lung tumor mass (2.8 cm × 2.1 cm) in the left upper lobe with pleural invasion.

**Figure 2:**
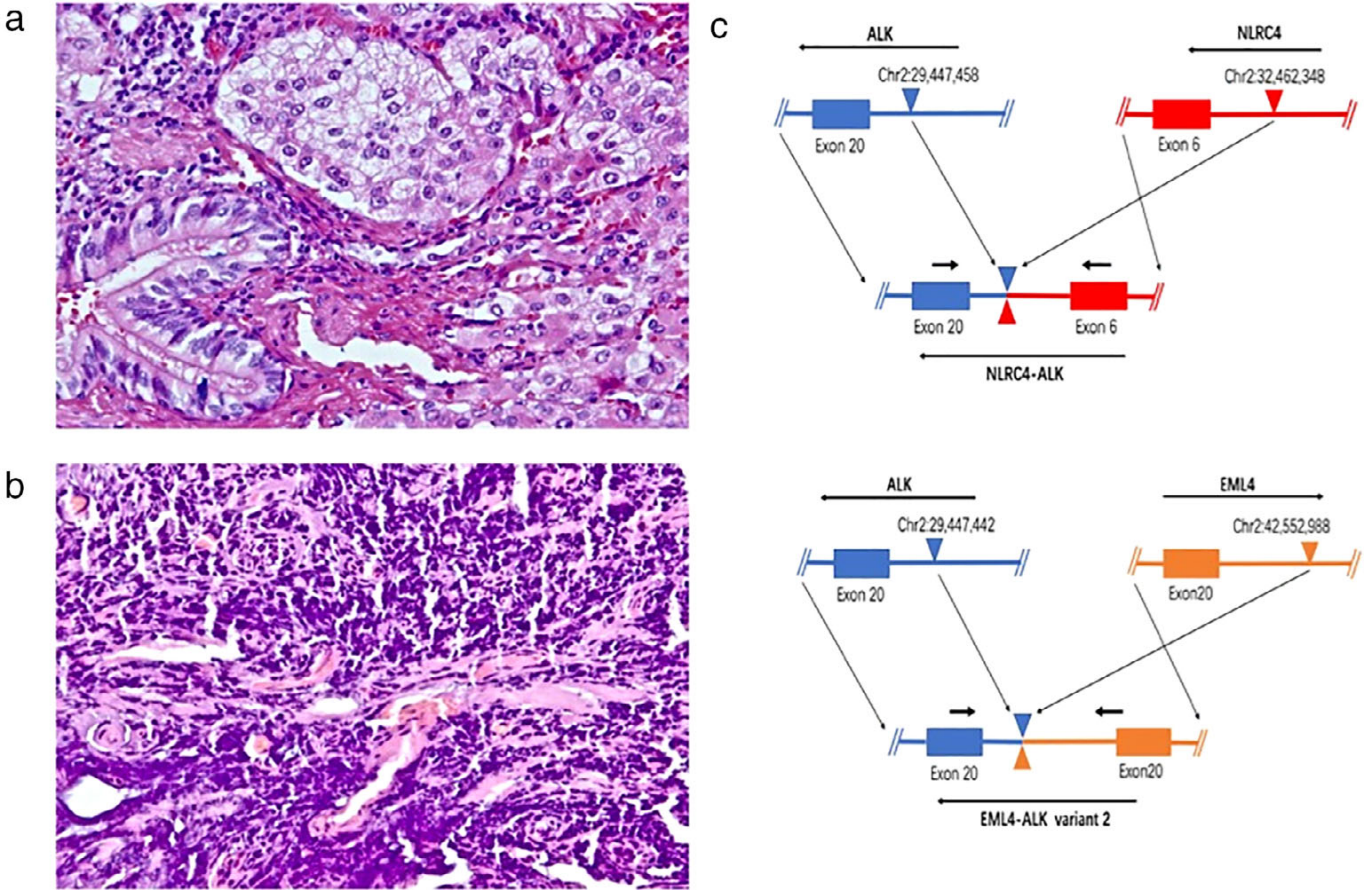
Histopathological findings and next‐generation sequencing (NGS) of the lung‐tumor‐tissue samples. (**a**) Primary lung adenocarcinoma was diagnosed (40×). (**b**) Pleural dissemination with nodules showed adenocarcinoma invasion (40×). (**c**) *ALK* gene and the *NLRC4* gene map to chromosome 2p, *NLRC4* is disrupted at a position, chr2 32 462 348 and is ligated to a position of chr2 29 447 458 of *ALK*, giving rise to the *NLRC4–ALK* fusion gene. *ALK* gene and the *EML4* gene map to chromosome 2p, but have opposite orientations. *EML4* is disrupted at a position, chr2 42 552 988 and is ligated to chr2 29 447 442 of *ALK*, giving rise to the *EML4–ALK* (variant 2) fusion gene.

In January 2019, a 64‐year‐old Chinese woman, who was a non‐smoker, was referred to our hospital because of patchy shadows in the left upper lung on chest X‐ray. Chest CT scan revealed a spiculated mass (2.8 cm × 2.1 cm) in the left upper lobe (Fig 1). She had no clinical symptoms of fever, cough, hemoptysis or dyspnea. Detection of serum tumor markers showed an increased level of cytokeratin 19 fragment (6.10 ng/mL; normal value, 0.00–3.00 ng/mL). The patient was assessed as being acceptable for surgery after head CT and bone single‐photon emission computed tomography (SPECT). On 21 February 2019, a pulmonary nodule (3.5 cm × 1.9 cm × 1.5 cm) and one of the pleural dissemination nodules (1 cm × 0.5 cm × 0.5 cm) were surgically removed. However, pleural effusion, pleural retraction and multiple implanted nodules were found during the operation. Postoperative pathology confirmed a stage IVa (pT2aN0M1a) ADC (Fig 2a,b).

To explore potential targeted therapies, next‐generation sequencing (NGS) was performed on postoperative pulmonary nodule specimen using a 56 cancer‐related gene panel. The coexistence of double ALK rearrangements were revealed, including an unreported *NLRC4‐ALK* (N6:A20) fusion and a *EML4‐ALK* (E20:A20, variant 2) fusion. In the novel *NLRC4‐ALK* rearrangement, the exon 6 of NLRC4 fused to the exon 20 of *ALK*, with an abundance of 24.44% and the fusion points were at chr2 32 462 348 and chr2 29 447 458. *EML4*‐*ALK* fusion was identified at an abundance of 15.33% (Fig 2c).

**Figure 3:**
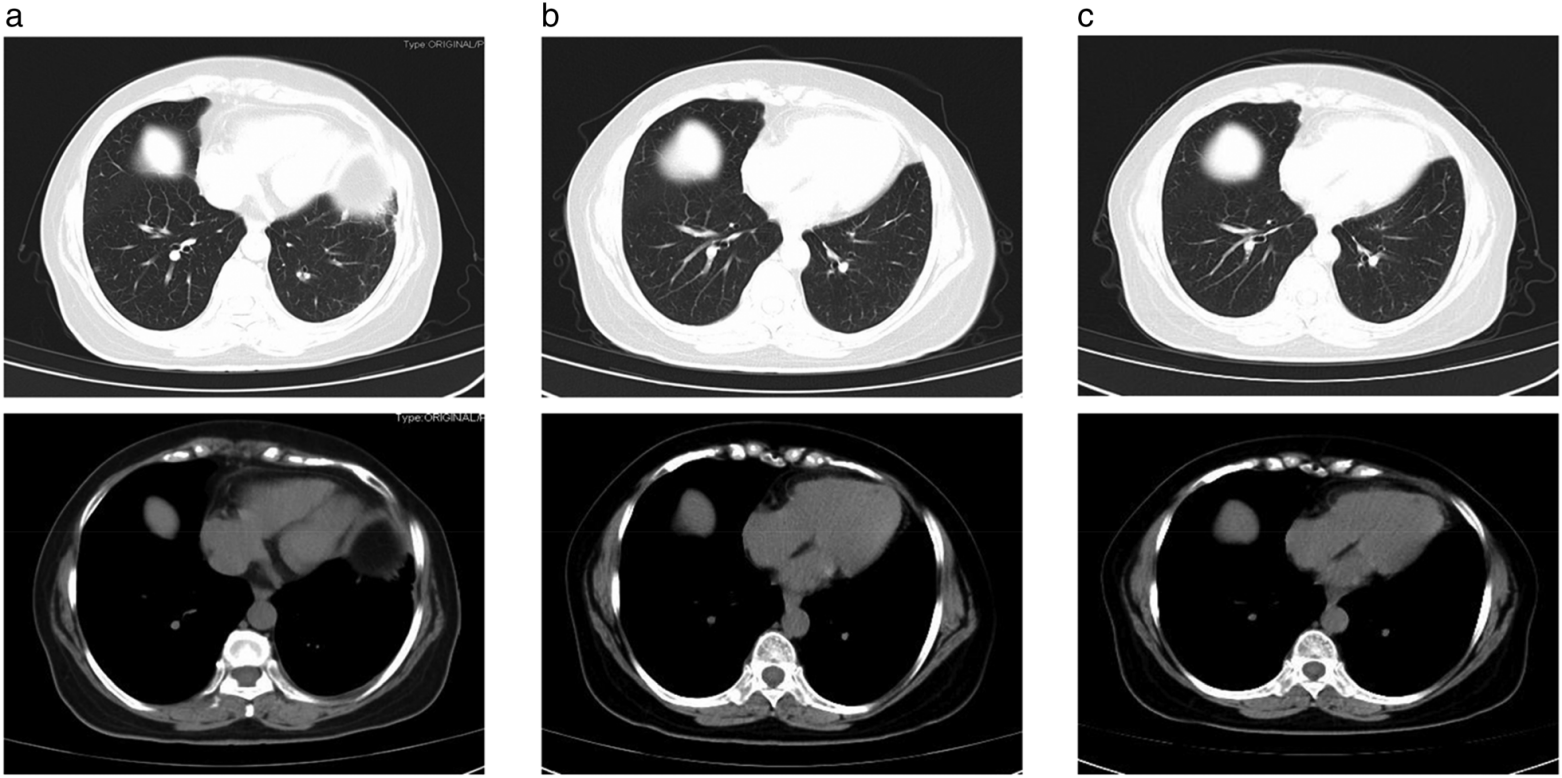
Subsequent chest CT scans performed (**a**) two, (**b**) seven and (**c**) 10 months after initiation of crizotinib treatment, and no signs of tumor recurrence were evident.

The patient received continuous oral crizotinib 250 mg twice daily as postoperative therapy from 10 March 2019, and no obvious drug‐related adverse effects were observed. Clinical and radiological follow‐up showed no evidence of recurrent (Fig 3a–c). To date, over 10 months after surgery, the patient still showed stable disease.

## Discussion

*ALK* gene arrangements are important driving oncogenes in NSCLC. Several different forms of *ALK* fusions have been reported, such as *EML4‐ALK*, the most common *ALK* fusion in NSCLC, which harbors the 5' end of *EML4* fused to the entire ALK kinase domain and leads to constitutive ligand‐independent kinase activation.^6^ However, *ALK* double fusions are rarely reported, and to our knowledge, only four cases have been previously reported, including *EML6‐ALK* and *FBXO11‐ALK*,^7^ *DYSF‐ALK* and *ITGAV‐ALK*,^8^ *EML4‐ALK* and *BCL11A‐ALK*,^9^ as well as *PRKCB‐ALK* and *EML4‐ALK*.^10^ In this report, we present the first case of novel *NLRC4‐ALK* and *EML4‐ALK* fusion mutations in ADC. When gene fusion happens, the expression of *ALK* kinase domain is regulated by the upstream regulatory element which derives from the fusion partner gene. Although there is no direct evidence to support *NLRC4‐ALK* as a driver mutation, considering that *NLRC4* has been reported to be highly expressed in lung tissues,^11^ there is a possibility that *NLRC4‐ALK* rearrangement is a driver mutation.

ALK‐TKIs have been widely used for ALK‐positive patients, but the responses are heterogeneous for patient with different *ALK* fusions.^12^ Especially, when two kinds of *ALK* mutations exist simultaneously in one patient, the effectiveness of ALK‐TKI treatment might be affected. In this case, the patient belonged to stage IV ADC accompanied with pleural metastasis; however, after 10 months of crizotinib treatment, no pleural dissemination was observed, which supported the effectiveness of crizotinib in patients with concomitant *NLRC4‐ALK* and *EML4‐ALK* mutations.

In conclusion, this report describes the first case of an ADC patient with an unreported *NLRC4‐ALK* fusion and *EML4‐ALK* fusion, with a PFS of over 10 months with continuous crizotinib treatment after surgery. Our report provides valuable information that patients with concurrent *ALK* double fusions could benefit from crizotinib, and provides a better understanding of ALK‐TKIs in ADC with *NLRC4‐ALK* rearrangement.

## Disclosure

No authors report any conflict of interest.
